# Insights into perfluorooctane sulfonate photodegradation in a catalyst-free aqueous solution

**DOI:** 10.1038/srep09353

**Published:** 2015-03-23

**Authors:** Xian-Jin Lyu, Wen-Wei Li, Paul K. S. Lam, Han-Qing Yu

**Affiliations:** 1 CAS Key Laboratory of Urban Pollutant Conversion, Department of Chemistry, University of Science and Technology of China, Hefei, China; 2 USTC-CityU joint Advanced Research Center, Suzhou, China; 3 State Key Laboratory in Marine Pollution, Department of Biology and Chemistry, City University of Hong Kong, Hong Kong SAR, China

## Abstract

Photodegradation in the absence of externally added chemicals could be an attractive solution for the removal of perfluorooctane sulfonate (PFOS) in aqueous environment, but the low decomposition rate presents a severe challenge and the underlying mechanisms are unclear. In this study, we demonstrated that PFOS could be effectively degraded in a catalyst-free aqueous solution via a reduction route. Under appropriate pH and temperature conditions, a rapid PFOS photodegradation, with a pseudo-first-order decomposition rate constant of 0.91 h^−1^, was achieved. In addition, hydrated electrons were considered to be the major photo-generated reductive species responsible for PFOS photodegradation in this system. Its production and reduction ability could be significantly affected by the environmental conditions such as pH, temperature and presence of oxidative species. This study gives insights into the PFOS photodegradation process and may provide useful information for developing catalyst-free photodegradation systems for decomposing PFOS and other persistent water contaminants.

Perfluorooctane sulfonate (PFOS) and perfluorooctanoate (PFOA) have been widely used in the industrial production sectors over decades, due to their excellent thermal, chemical stability and surface activity^1^. However, they are also environmentally persistent and bio-accumulative^2^,^3^, and have high developmental toxicity, immunotoxicity, and hepatotoxicity^4^,^5^,^6^. With a growing awareness of the hazards of these compounds, their production and application have been restricted by some countries so far^1^,^3^. Nevertheless, in many countries they are still being manufactured and extensively used^7^, resulting in still high environmental concentration^7^ and even raising concentration in some regions^8^. Thus, effective approaches for degradation of these compounds are needed.

The PFOS/PFOA degradation involves the cleavage of carbon-fluorine bonds, which requires advanced treatment approaches, such as photochemical^9^,^10^,^11^,^12^,^13^, sonochemical^14^, subcritical^15^, microwave-hydrothermal^16^, electrochemical^17^ and plasma chemical degradation^18^. Amongst these, the photodegradation process has drawn great interest due to its operational simplicity and the potential to directly utilize the inexhaustible solar energy. Compared with PFOA, PFOS is more difficult to degrade due to the presence of ionic headgroup^9^,^15^, In existing photochemical PFOS degradation systems, extra chemicals, e.g., alcohol^10^,^12^, ferric ion^13^, iodide or persulfate^19^ were usually added to allow an efficient degradation, but this also increases the costs and arouses environmental concerns. Therefore, it is interesting to know whether an efficient photodegradation of PFOS could also be achieved in a catalyst-free system (i.e., without addition of extra chemicals) and how this process occurs.

This study aims to shed light on the photodegradation process of PFOS in a catalyst-free aqueous solution and elucidate the underlying mechanisms. The roles of water photolysis-derived oxidative or reductive species in PFOS degradation were investigated by selectively adding specific promoters or inhibitors, such as oxygen (O_2_), hydrogen peroxide (H_2_O_2_), nitrous oxide (N_2_O), tert-butanol (t-BuOH), or by adjusting the pH and temperature. The findings in this study may provide useful information and guidance for future optimization of PFOS photodegradation processes.

## Results

### Photodegradation of aqueous PFOS

**Figure 1:**
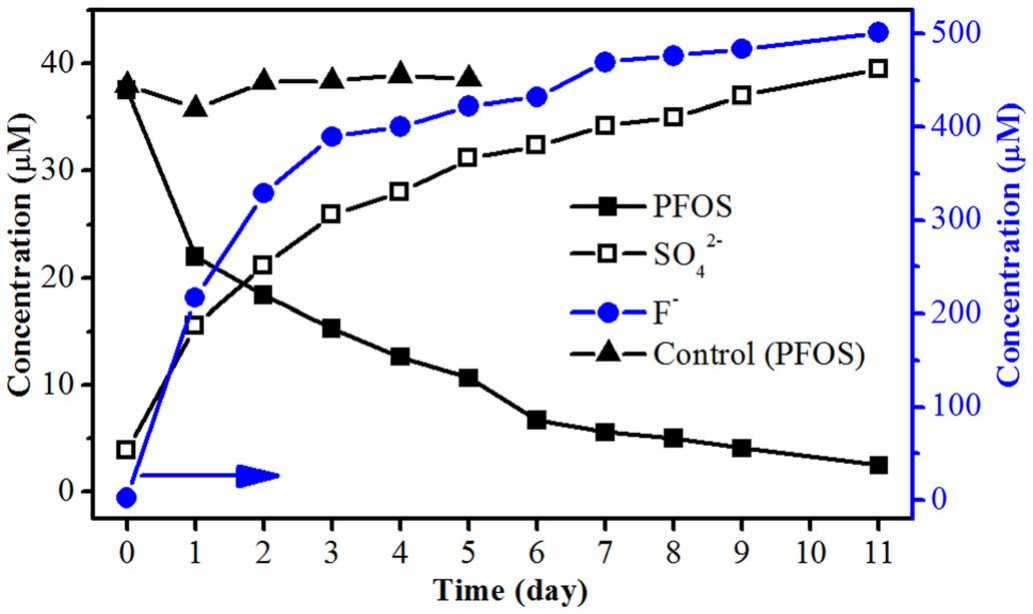
Concentrations of PFOS, fluoride ions, and sulfate ions during photodegradation (11-day) in unbuffered aqueous solution in Reactor B. Initial pH: 7.4, final pH: 3.5, temperature: ~90°C. The control experiment was conducted under the same condition but without UV irradiation, and its final pH was 7.6.

The photodegradation of neat aqueous PFOS under UV irradiation of medium pressure mercury lamp (500 W) was investigated. Figure 1 shows a gradual decline of PFOS concentration over time, accompanied by increased fluoride and sulfate ions. This result confirmed that the carbon-fluorine and carbon-sulfur bonds of PFOS, despite their weak light absorbance (Figure S1), were broken to form sulfate and fluoride ions under UV irradiation. In addition, no degradation of PFOS was observed after 6-h irradiation when a xenon lamp (500 W) was used as the light source. Thus, it could be inferred that UVC band (λ: 100–280 nm) should be responsible for the PFOS photodegradation in the medium pressure mercury lamp system. In all the samples, the mole ratios of the formed sulfate ions to the decomposed PFOS ranged from 0.98 to 1.16, and the defluorination ratios were between 0.82 and 1.03. All these results suggest a high decomposition degree of PFOS. Nevertheless, the complete degradation of 37.2 μM PFOS under the present conditions took more than 11 days.

Here, a powerful light source, with a UVC intensity (λ ≤ 290 nm, mainly around 254 nm) of 20 mW·cm^−2^ according to chemical actinometry (iodide/iodate)^20^, was used. However, the degradation rate (*k* = 0.012 h^−1^, adjusted *R*^2^ = 0.9503) was not significantly increased compared to a previous study (*k* = 0.0054 h^−1^) with a much lower UV intensity (λ = 254 nm) of 3.73 mW·cm^−2^^10^, indicating that light intensity might not be a limiting factor in our system.

Photodegradation of organic compounds can take place through direct and/or indirect photolysis. According to Eq. 1, both oxidative species (e.g., hydroxyl radicals ·OH), and reductive species (e.g., hydrogen atoms (H·) and hydrated electrons (e_aq_^−^)), could be generated from water splitting under UV irradiation^21^.
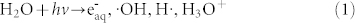


The presence of these species in irradiated solution was confirmed by the electron paramagnetic resonance analysis (Figure S2). Photodegradation of PFOS might be attributed to direct photolysis and/or an attack by these aggressive species. To clarify this, we tested the PFOS decomposition rates under specifically controlled oxidative or reductive conditions.

We first investigated the PFOS degradation in a non-buffered aqueous solution, and found a substantial decrease in the solution pH from 7.4 to 3.5 during 6-h reaction (Figure S3). This should be attributed to the formation of acidic compounds such as hydrofluoric acid (HF, p*K*_a_ = 3.14^22^) and hydrosulfate (HSO_4_^−^, p*K*_a_ = 1.99^22^) during PFOS degradation. For instance, 5% of PFOS (37.2 μM) mineralization would reduce the pH value from neutral to about 4.5. Thus, in order to eliminate the pH impact on PFOS degradation, 6.0 mM phosphate buffer solution (PBS) was used in the subsequent experiments. A significant increase in the PFOS decomposition rate (*k* = 0.13 h^−1^, adjusted *R*^2^ = 0.9999) was observed (Figure S4). Although PBS had an intense absorbance in UVC band (λ ≤ 220 nm, Figure S1), the enhanced degradation was likely associated mainly with the stable pH rather than the phosphate-induced adsorption (discussed below).

### PFOS resistance to oxidative degradation

To validate the possible role of oxidative species in PFOS photodegradation, an oxidative solution environment was created by purging O_2_ or dosing with H_2_O_2_. Notably, although O_2_ and H_2_O_2_ will quench some radicals, such as hydroxyl radicals, UV/O_2_ and UV/H_2_O_2_ systems can provide more reactive oxygen species (ROS) than UV alone system. In the oxygenation test, O_2_ was continuously supplied to maintain a high dissolved oxygen level (0.81 mM, estimated according to a O_2_ partial pressure of 101 kPa at 90°C)^23^. In another test, 30% (w/w) H_2_O_2_ solution was continuously dosed at a rate of 1.0 mL·h^−1^. Thus, given the H_2_O_2_ decomposition under UV irradiation was compensated by the continuous H_2_O_2_ addition, the effective concentration of H_2_O_2_ was estimated to be 8.8 mM.

**Figure 2:**
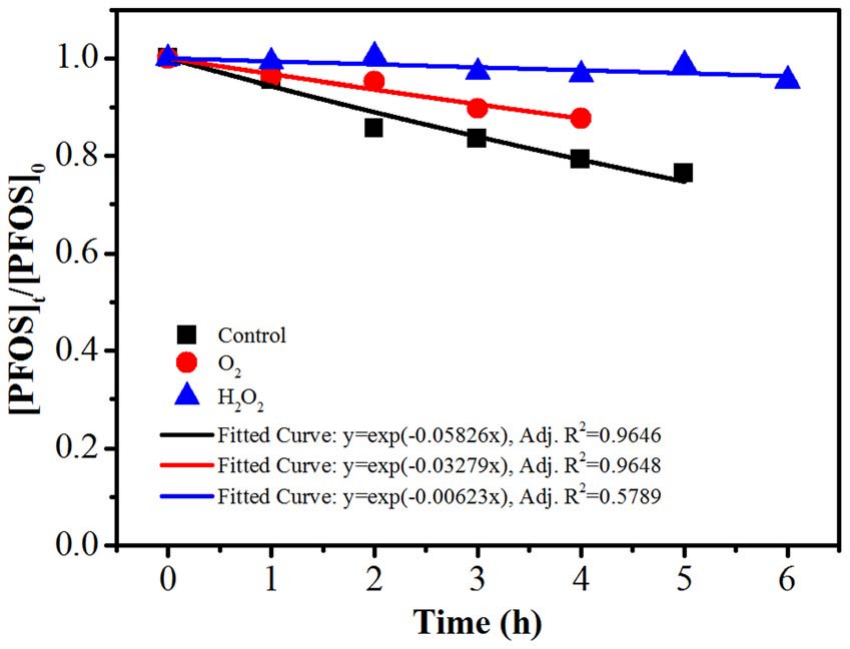
The time profiles of PFOS (37.2 μM) decomposition and the fitted curves when O_2_ or H_2_O_2_ was introduced. PBS: 6.0 mM (pH 7.0), [O_2_] = 0.81 mM, [H_2_O_2_] = 8.8 mM, temperature: 90°C. The control experiment was conducted under the same condition but without extra chemical additions.

The PFOS decomposition profiles and the fitted results under the above conditions are shown in Figure 2. The PFOS photodegradation was substantially suppressed under oxygenated conditions (*k* = 0.033 h^−1^) and even more so under the H_2_O_2_-dosing condition (*k* = 0.0062 h^−1^) compared to the control (*k* = 0.058 h^−1^). Apparently, the oxidative species generated in the presence of O_2_ or H_2_O_2_ were ineffective and even detrimental to the PFOS photodegradation. This is consistent with a previous report that hydroxyl radicals could not decompose PFOS under relatively mild conditions^14^. Therefore, reductive degradation should be the main route of PFOS decomposition in our system, which process could be considerably impaired by the presence of oxidative species. On one hand, O_2_, H_2_O_2_ and the generated ROS might directly quench some photo-generated reactive species. On the other hand, a fraction of the UV photons might be absorbed by O_2_ or H_2_O_2_ (Figure S1)^24^, reducing the available photons for PFOS decomposition.

### PFOS photodegradation attributable to reduction

To validate the above hypothesized mechanism, the degradation of PFOS in the presence of N_2_O or t-BuOH was investigated. N_2_O can convert hydrated electrons (Eq. 2) or hydrogen atoms (Eq. 3) to hydroxyl radicals^25^,^26^, while t-BuOH can convert hydroxyl radicals to the less reactive radicals (Eq. 4) and react with hydrogen atoms with a low rate constant (Eq. 5)^25^,^27^.
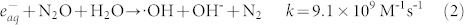

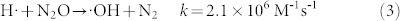

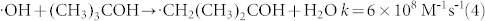

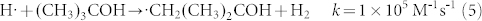


**Figure 3:**
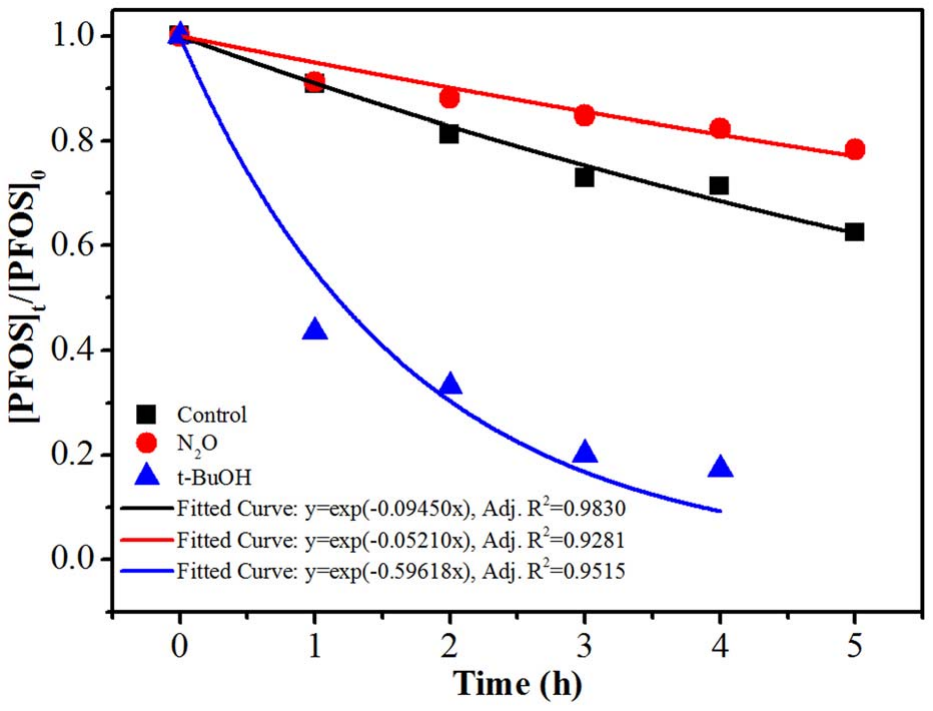
The time profiles of PFOS (37.2 μM) decomposition and the fitted curves in 6.0 mM PBS (pH 7.0) with addition of either N_2_O or t-BuOH. [N_2_O] = 8.4 mM, [t-BuOH]: 1.0 mM, temperature: 100°C. The control experiment was conducted under the same condition but without extra chemical additions.

Figure 3 illustrates the PFOS decomposition rate profiles and fitted results in the presence or absence of different scavengers. As expected, N_2_O (8.4 mM, calculated assuming the N_2_O partial pressure of 101 kPa and according to its solubility at 80°C)^28^ significantly lowered the decomposition rate (*k* = 0.052 h^−1^) compared with the control (*k* = 0.094 h^−1^), confirming that PFOS photodegradation could be suppressed by the formed hydroxyl radicals^26^. Meanwhile, the addition of t-BuOH (1.0 mM) drastically increased the degradation rate (*k* = 0.60 h^−1^). All these evidences suggest that PFOS in the aqueous solution was mainly subjected to reductive photodegradation.

### Effects of solution pH and temperature

**Figure 4:**
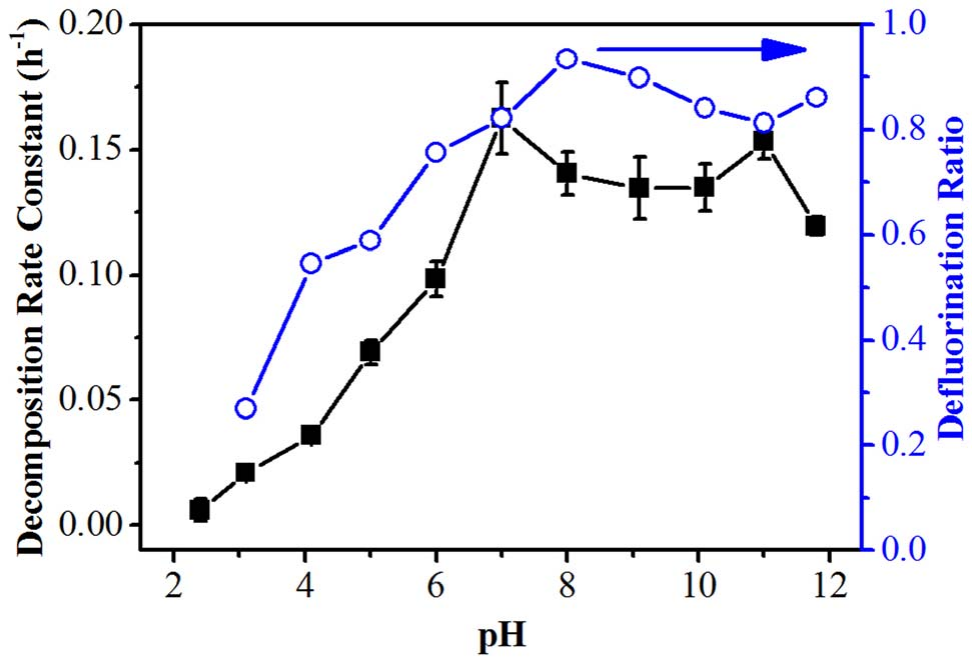
The PFOS (37.2 μM) decomposition rate constants and defluorination ratios (6-h reaction) at different initial pH values in Reactor B. PBS: 6.0 mM, temperature: ~90°C.

**Figure 5:**
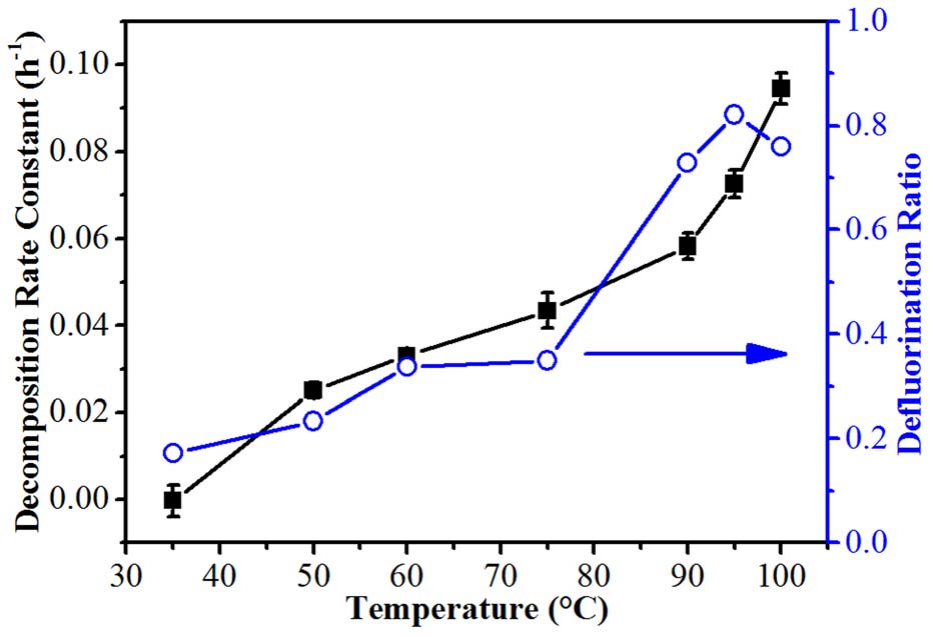
The PFOS (37.2 μM) decomposition rate constants and defluorination ratios (5-h reaction) at different temperatures and fixed pH of 7.0 (6.0 mM PBS).

The above conclusion was also supported by the results obtained under different pH and temperature conditions, because both factors could influence the production and consumption of reductive species and hence lead to different PFOS degradation performances. An investigation of the photodegradation at different pHs shows that the PFOS decomposition rates and defluorination ratios decreased at a lower pH, and almost no decomposition was observed at pH 2.4 after 6-h irradiation (Figure 4 and Table S1). Thus, the rapid decrease in pH might be a critical reason responsible for the slow PFOS degradation in the non-buffered solution. Considering, the low p*K*a value of PFOS (−3.27), direct photolysis of PFOS was unlikely to occur in our system, while photoreductive decomposition should be the only route. In addition, as shown in Figure 5, a positive correlation between the PFOS photodegradation rate and the solution temperature (from 35 to 100°C) was observed (also see Table S2 for more details). The activation energy for the initial defluorination of PFOS in the PBS was 25 ± 2 kJ/mol, with an Arrhenius pre-exponential factor A of 272 h^−1^ (Figure S5). Here, the decomposition rate constants (in Reactor A) were slightly lower than those in the pH tests (in Reactor B) despite of the same pH. This should be due to the different average irradiation intensities in the different-sized reactors.

Thus, a higher level of reductive species in the solution and accelerated PFOS degradation might be encouraged by the raised pH and temperature. The underlying mechanism is detailed below.

## Discussion

Photodegradation without chemical-dosing in aqueous solution is a promising process to treatment PFOS, but a low degradation efficiency presents a severe limitation at the present stage^10^,^13^. This study attempts to find out the reason behind such a slow PFOS degradation. The results indicate that the PFOS photodegradation in a catalyst-free aqueous solution was a reduction process, in which reductive species such as hydrogen atoms and/or hydrated electrons from water photolysis play important roles. The production of these reductive species could be significantly affected by solution pH and temperature. According to Eq. 6, the high-concentration hydroniums (H_3_O^+^) in acidic solution would quench the hydrated electrons to form hydrogen atoms. Thus, the suppressed degradation at acidic pH suggests that hydrated electrons, rather than hydrogen atoms, might be the major contributors for the PFOS degradation. In addition, the thermodynamic analysis also indicates that the hydrogen atoms (standard reduction potential *E*° = −2.1 ~ −2.3 V)^25^ could not break the primary and secondary carbon-fluorine bonds of PFOS (*E* ≤ −2.7 V)^1^, while hydrated electrons could (*E*° = −2.7 ~ −2.9 V)^25^. A higher temperature tend to increase the hydrated electrons generation and thus also favor PFOS degradation. Therefore, hydrated electrons should be the key reductive species responsible for the PFOS photodegradation in the present study, although more direct evidences are still to be provided.
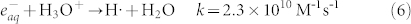


Based on the above analysis, we hypothesize the following mechanism for the PFOS photodegradation in a simple aqueous solution. Firstly, hydrated electrons are generated from water photolysis (Eq. 1) and/or the charge-transfer-to-solvent reaction of hydroxide ions (Eq. 7)^29^. A fraction of these generated hydrated electrons are immediately quenched via the recombination with the concurrently photo-generated hydroxyl radicals, H_3_O^+^, and even water molecules, while another fraction reduces PFOS. Then, the reduction products of PFOS, (i.e., after defluorination and/or desulfonation), which are less persistent owing to lower fluorination nature of the ionic headgroup, undergo rapid further degradation (photolysis, oxidation and/or reduction) and mineralization^9^,^30^. In this complicated reaction process, there might be some other degradation products, such as formic acid and fluorine-contained metabolites^9^,^10^,^30^. Especially, short-chain perfluorocarboxylic acids, namely, C_n_F_2n+1_COO^−^ (n = 6 and 7), were expected to form during PFOS photodegradation^10^. However, they were not detected in our study, probably due to their rapid decomposition under the tested conditions. For instance, PFOA (C_7_F_15_COO^−^) decomposed at a high rate of 2.78 h^−1^ at pH 7.0 and 90°C (Figure S6), while PFOS at 0.058 h^−1^.
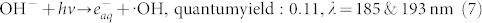


Notably, defluorination ratio under this chemical-free conditions (typically > 70%) was higher than under iodide-dosing conditions^9^, probably because the hydroxyl radicals generated along with the hydrated electrons (Eq. 7) in our study were also involved in the subsequent processes to yield fluoride^30^,^31^.

**Figure 6:**
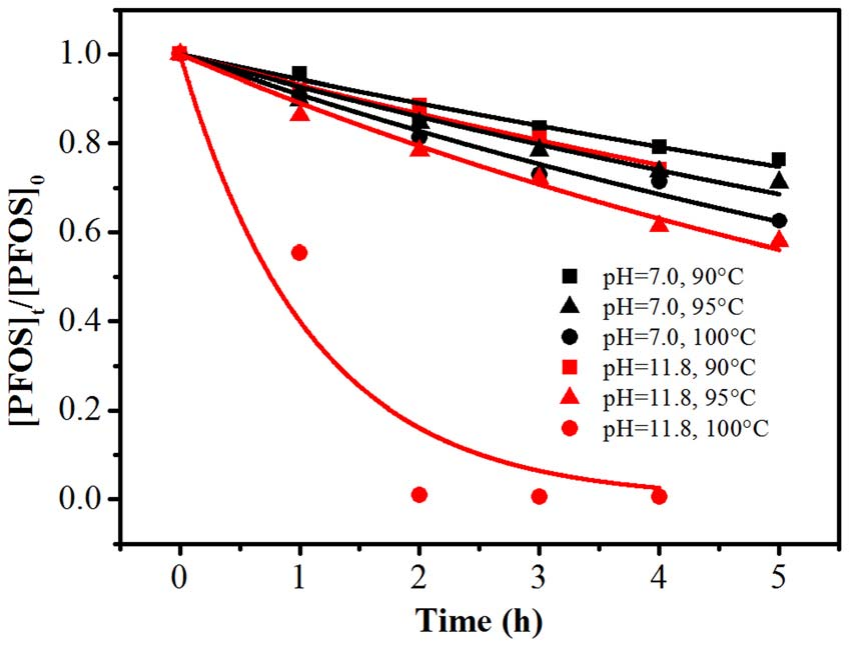
The time profiles of PFOS (37.2 μM) decomposition and the fitted curves under simultaneously altered pH and solution temperature (6.0 mM PBS).

With the deep understanding of the catalyst-free PFOS photodegradation, an efficient photodegradation of aqueous PFOS may be enabled by enforcing appropriate pH, temperature, and anoxic conditions. To optimize this process, we explored the PFOS decomposition under different pH and temperature combinations. Figure 6 shows that the decomposition rate was only slightly increased when the temperature was raised from 90 to 100°C at pH 7.0 (see Table S2 for more details). However, a significantly increased degradation was observed at pH 11.8. In contrast, no degradation was observed in the absence of UV irradiation. Thus, by applying an alkaline pH of 11.8 and maintaining the solution temperature at 100°C, a very high decomposition rate constant of 0.91 h^−1^ was achieved. This performance is superior to most chemical-dosed systems (Table 1), indicating that that an efficient photodegradation can also be achieved in catalyst-free system.

Nevertheless, such high temperature and alkalinity conditions might be unrealistic for practical PFOS degradation applications. To make it a viable technology for PFOS elimination, other efficient and low-cost strategies for enhancing the generation of hydrated electrons should be developed. For example, it might be preferable to utilize solar energy for driving the in-situ PFOS degradation^12^, and to take advantage of some natural contaminants that may strengthen the generation of hydrated electrons^32^.

In summary, this study demonstrates the possibility of efficient photodegradation of PFOS in a simple aqueous solution and partially elucidates the underlying mechanism. Reduction by hydrated electrons was considered to be the major route for PFOS photodegradation in this system, and its production could be controlled by adjusting the environmental conditions such as anoxic condition, pH and temperature. This study deepens our understanding of the PFOS photodegradation process and may offer implications for optimized design and operation of catalyst-free photodegradation systems for decomposing PFOS and other persistent water contaminants.

## Methods

### Chemicals and reagents

PFOS potassium salt (>98.0%) was purchased from Tokyo Chemical Industry Co., China, while methanol (HPLC grade) was purchased from Sigma-Aldrich Co., USA. All the other chemicals (AR grade) were obtained from Sinopharm Chemical Reagent Co., China. Deionized water was prepared with a Milli-Q Gradient water purification system (Millipore Inc., USA).

Stock solution of PFOS (37.2 μM) was prepared by directly dissolving 80.0 mg PFOS potassium salt into 4.0 L deionized water. In the experiments, various chemicals were directly added into the PFOS stock solution to prepare the reaction solutions.

### Photodegradation experiments

Two photoreactors, both equipped with a medium pressure mercury lamp (500 W, GY-500, Beijing Tianmaihenghui Co., China) as UV irradiation source (emission is shown in Figure S7), were used in the PFOS photodegradation tests. Reactor A (Figure S8) had a quartz tube insider for inner UV irradiation and was semi-sealed at the top (connected to outside through a condenser and a 0.45 μm mixed cellular fiber filter), and it was used for all the tests except for long-time scale (≥24 h) reactions and the study on pH effects. The reactor was fed with 1000 mL reaction solution, stirred by a magnetic stirrer at 120 rpm, and irradiated internally with the mercury lamp. Due to the significant heat generation of the lamp (the medium pressure mercury lamp usually has a low UV efficiency, typically less than 28%)^33^, the reaction solution could be warmed up by the lamp. To resolve this problem, a water bath was used to control the temperature of the solution, which was monitored using a thermometer. Reactor B was made of a double layered quartz tube (semi-sealed as Reactor A), but without stirring and temperature monitoring. It was smaller and fed with 200 mL reaction solution. Due to a relatively slow heat exchange, the temperature of the solution was maintained at about 90°C (measured using a thermometer after the experiment) during the UV irradiation, with the assistance of a water bath.

The deoxygenated (purging nitrogen for 10 min) PFOS solution was irradiated for 4–6 h and the solution (2 mL) was sampled on an hourly basis using a syringe, while a longer irradiation time (2–11 d) was adopted in our preliminary experiments. Although the reaction solutions were hot in most of the testş the evaporation loss was minimal (≤5%) due to the semi-closed system. According to the experimental design, O_2_ or N_2_O was pumped into the reactor from the bottom of the reaction solution at 20 mL/min. Phosphate (6.0 mM) was employed as the pH buffer to eliminate the impact of varying pH, and the initial pH of the solution was adjusted using 10 M KOH (for pH ≤ 4, using 6.0 mM H_3_PO_4_ and KOH; for 5 ≤ pH ≤ 11, KH_2_PO_4_ and KOH; for pH = 11.8, K_3_PO_4_ only). In case of H_2_O_2_ or t-BuOH addition, these chemicals were added into the solution prior to pH adjustment.

### Analysis

PFOS concentration was measured using a high performance liquid chromatograph (HPLC, 2695, Waters Inc., USA) coupled with a mass spectrometer (MS, LCQ Advantage MAX. Thermo Fisher Scientific Inc., USA). Prior to the measurement, the sample was diluted with methanol to ensure a PFOS concentration of less than 2.5 μM. In addition, PFOS standard solutions of 0.2–2.5 μM were prepared in order to generate the external calibration curves. Blank and two standard solutions (0.2 and 2.5 μM) were also analyzed in each sample sequence in accordance with the order from low to high concentrations. A Phenomenex Luna 5 μ C18(2) 100A separation column (4.6 mm i.d. × 150 mm, 5 μm particles) was used for the analysis. The column temperature was set at 40°C. A mixture of eluent A (10 mM ammonium acetate in water) and B (methanol) was employed as the mobile phase, and the flow rate was maintained at 0.5 mL/min. The eluent gradient started with 40% B for 1 min and then was lineally increased to 100% B within 6 min and held at that gradient for 5 min, and eventually returned to the starting conditions within 1 min and held for 2 min for equilibrium during the injection interval. The MS detection was operated in a negative mode by using an electrospray ionization source. In order to maximize the transmission of PFOS anion (m/z 499), the following instrument parameters were adopted: nitrogen sheath gas flow rate, 25 arbitrary units; aux/sweep gas flow rate, 3 arbitrary units; spray voltage, 4.5 kV; heated capillary temperature, 350°C; capillary voltage, −4 V; and tube lens offset, 10 V.

The fluoride and sulfate ions were measured using an ion chromatograph (ICS-2000, Dionex Co., USA), which consisted of a degasser, a guard column (IonPac AG11-HC, 4 × 50 mm), a separation column (IonPac AS11-HC, 4 × 250 mm), a column heater (30°C), and a conductivity detector with a suppressor. An online generated KOH solution (30 mM), with a flow rate of 1.5 mL/min, was used as the mobile phase. The suppresser current was set at 112 mA. The aqueous standard solutions containing both fluoride (0–1000 μM) and sulfate (0–100 μM) were prepared to produce the external calibration curves. Blank and all the standard solutions were analyzed in the beginning of each sample sequence.

The solution pH was measured with a pH meter (Delta 320, Mettler Toledo Co., USA) at ambient temperature (20°C).

### Calculations

The PFOS decomposition rate constants were calculated assuming that the photodegradation followed pseudo-first-order decay kinetics (Eq. 8)^9^,^10^:
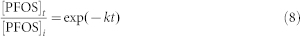
where [PFOS]*_i_* and [PFOS]*_t_* are the PFOS concentrations at the start of the reaction and at the irradiation time *t*, respectively, and *k* is the PFOS decomposition rate constant. Although pseudo-first-order kinetics might not be very suitable for some cases with significantly varied pH, our data show that one of the reactants (i.e., hydrated electrons) remained a constant concentration during the reaction process, suggesting that is appropriate and acceptable to adopting the pseudo-first-order kinetic constant for a quantitatively evaluation of the PFOS decomposition in this study.

In addition, the degree of the PFOS decomposition can be reflected by the defluorination ratio, which is defined as (Eq. 9):
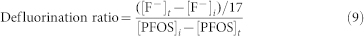
where [F^−^]*_i_* and [F^−^]*_t_* refer to the initial concentration of fluoride ions and their concentration at time *t*, respectively.

## Author Contributions

X.J.L. conducted the experiment the experiment and carried out data analysis. X.J.L., W.W.L., P.K.S.L. and H.Q.Y. designed the experiments. X.J.L. and W.W.L. wrote the manuscript.

## Supplementary Material

Supplementary Information Supplementary information

## Figures and Tables

**Table 1:** Comparison of this study with reported methods for PFOS degradation

Method	Conditions	*k^a^* (h^−1^)	*E*_EO_^b^ (10^3^ kWh/m^3^/order)	Reference
Direct UV	[PFOS]: 40 μM	0.0054	18.19	Yamamoto, et al.^10^
	750 mL			
	36–46°C			
	LPML^c^: 32 W			
UV in iso-propanol	[PFOS]: 40 μM	0.039	2.52	Yamamoto, et al.^10^
	[NaOH]: 68 mM			
	750 mL iso-propanol			
	38–50°C			
	LPML^c^: 32 W			
UV/KI	[PFOS]: 20 μM	0.18	3.41	Park, et al.^19^
	[KI]: 10 mM			
	30 mL			
	ambient temperature			
	UV: 8 W			
UV/K_2_S_2_O_8_	[PFOS]: 20 μM	0.24	2.56	Park, et al.^19^
	[K_2_S_2_O_8_]: 10 mM			
	30 mL			
	ambient temperature			
	UV: 8 W			
UV/FeCl_3_	[PFOS]: 20 μM	0.070	1.90	Jin, et al.^13^
	[FeCl_3_]: 100 μM			
	400 mL			
	25°C			
	LPML^c^: 23 W			
Sonolysis	[PFOS]: 20 μM	0.96	8.00	Moriwaki, et al.^14^
	60 mL			
	20°C			
	ultrasonic: 200 W, 200 kHz			
Plasma bubble	[PFOS]: 100 μM	0.15	3.99	Yasuoka, et al.^18^
	50 mL			
	25°C			
	Power: ~13 W (oxygen plasma)			
UV with optimization of pH and temperature	[PFOS]: 37.2 μM	0.91	1.27	this work
	PBS: 6.0 mM, pH 11.8			
	100°C			
	1000 mL			
	MPML^d^: 500 W			

^a^pseudo-first-order rate constants;

^b^electrical energy per order, defined as the number of kilowatt hours of electrical energy required to reduce the concentration of a pollutant by 1 order of magnitude in 1 m^3^ of contaminated water, proposed as figure-of-merit for removal of pollutant at low concentrations by the Photochemistry Commission of the International Union of Pure and Applied Chemistry^34^;

^c^low pressure mercury lamp;

^d^medium pressure mercury lamp with a low UVC luminous efficiency, which also acted as the heat source.
