# Supplementary material for: Insights into perfluorooctane sulfonate photodegradation in a catalyst-free aqueous solution
Source: Sci Rep. 2015 Mar 23;5:9353. doi: 10.1038/srep09353 (PMC4399502; doi:10.1038/srep09353)
Supplement: Supplementary Information [file srep09353-s1.doc]

**Supplementary information for**

**Insights into perfluorooctane sulfonate photodegradation in a catalyst-free aqueous solution**

Xian-Jin Lyu^1,2,3^, Wen-Wei Li^1,2,*^, Paul K.S. Lam^2,3,*^, Han-Qing Yu^1,2^

^1^CAS Key Laboratory of Urban Pollutant Conversion, Department of Chemistry, University of Science and Technology of China, Hefei, China

^2^USTC-CityU joint Advanced Research Center, Suzhou, China

^3^State Key Laboratory in Marine Pollution, Department of Biology and Chemistry, City University of Hong Kong, Hong Kong SAR, China

^*^**Corresponding authors**:

Dr. Wen-Wei Li, Tel: +86 551 63607592, Fax: +86 551 63601592, E-mail: [wwli@ustc.edu.cn](mailto:wwli@ustc.edu.cn); Prof. Paul K.S. Lam, Tel: +852 34427681, Fax: +852 34420522, E-mail: [bhpksl@cityu.edu.hk](mailto:bhpksl@cityu.edu.hk)


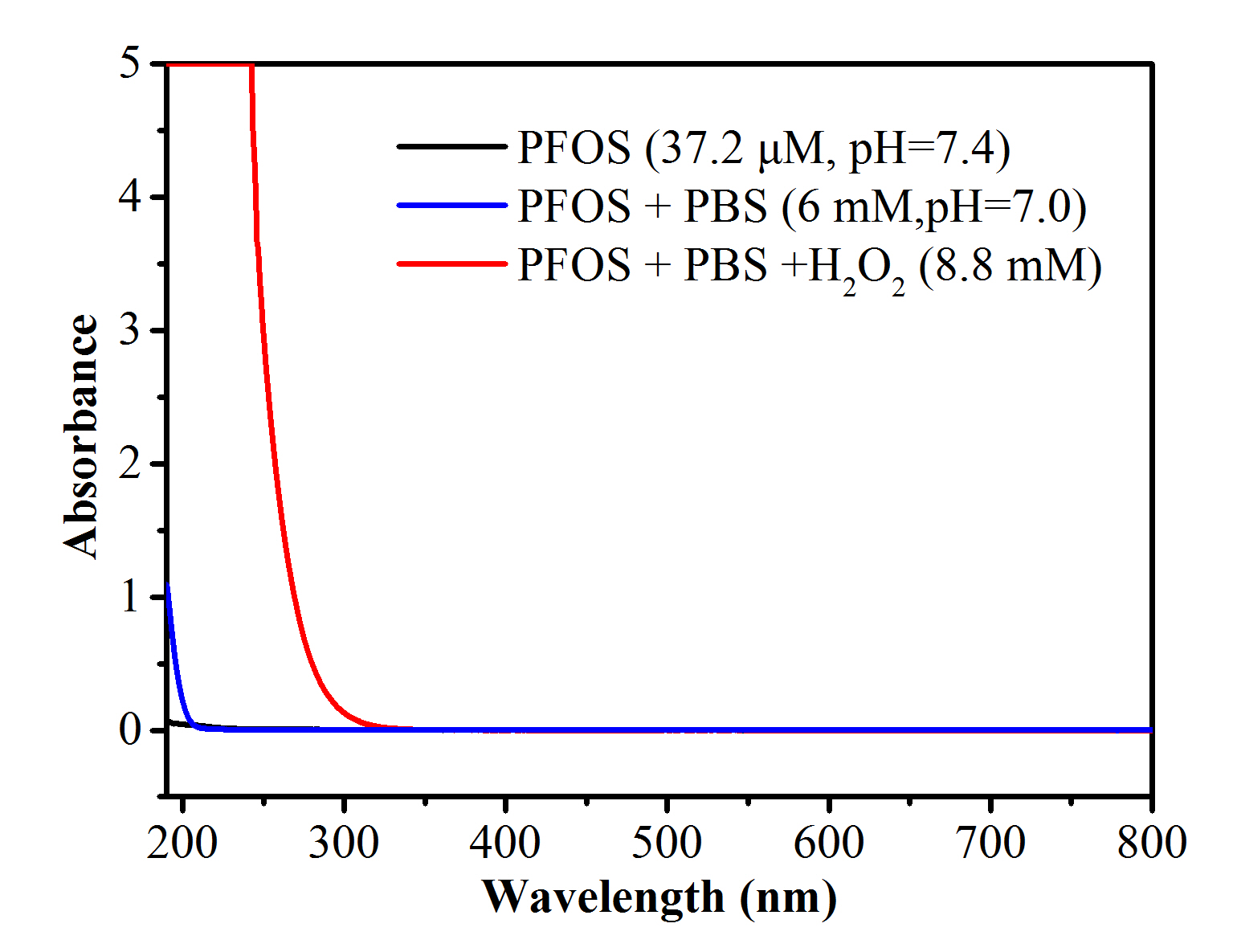


**Figure S1**. UV-Vis absorbance spectra of the PFOS (37.2 μM) different solutions. UV-Vis Spectrophotometer: UV-2550 (Shimadzu Co., Japan)

**Figure S2**. X-band electron paramagnetic resonance (EPR) spectrum of aqueous PFOS (185 μM) solution at ambient temperature (20 °C) after 10-second UV irradiation. EPR experiment was conducted with JES-FA200 spectrometer (JEOL, Japan), and the settings for the ESR spectrometer were as follow: center field = 323.97 mT; sweep width = 10 mT; microwave frequency = 9.08 GHz; modulation frequency = 100 kHz and power = 3.0 mW. 5,5-dimethyl-1-pyrroline N-oxide (DMPO) was used as the spin-trapping reagent at a concentration of 0.1 M. Asterisks denote the peaks of •DMPO–OH adduct, which could be arise from the trapping of a hydroxyl radical, and circle denote the peaks of •DMPO–H adduct, which could be arise from the trapping of a hydrogen atom and/or by the reaction of the hydrated electron^1,2^.


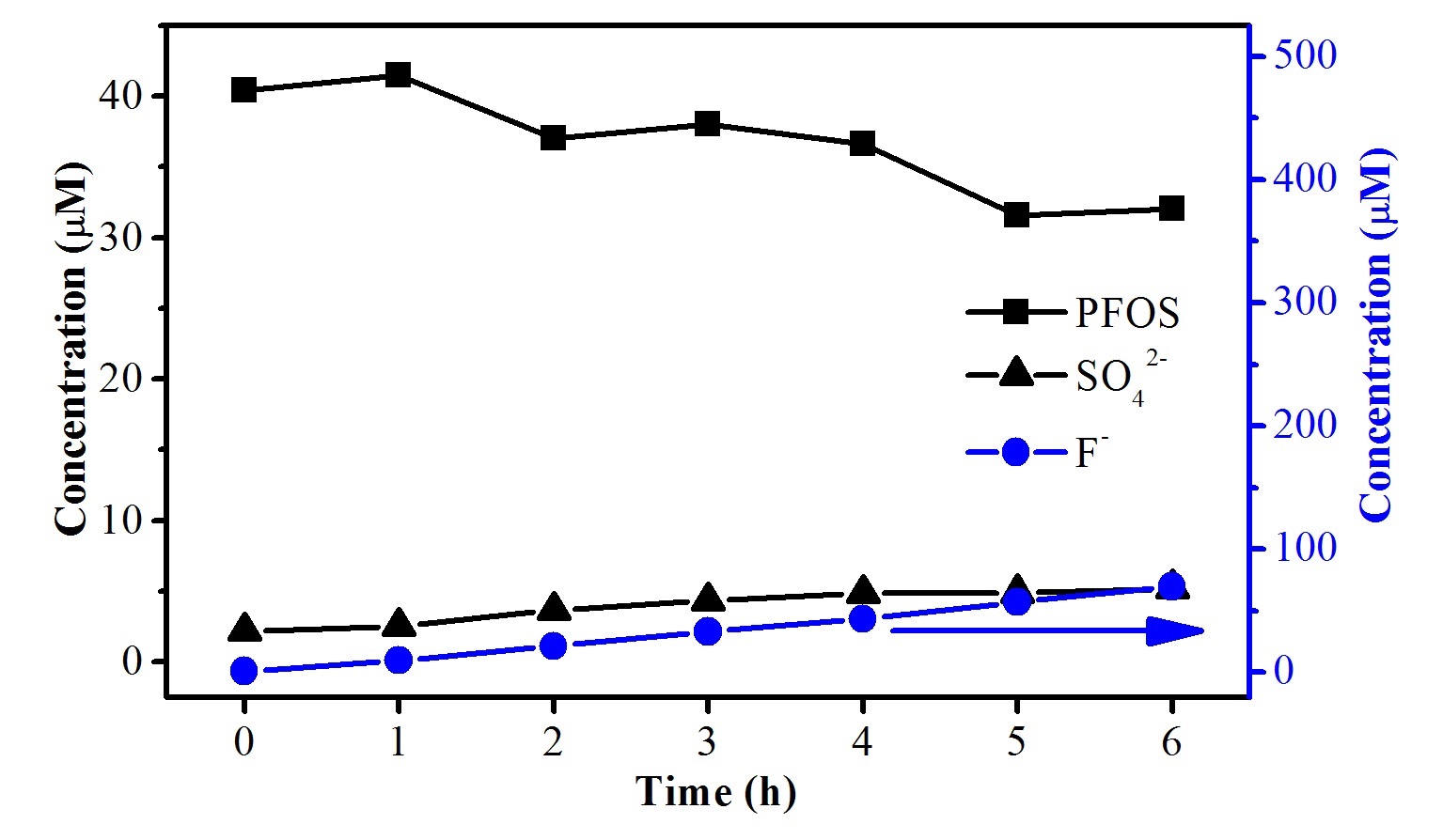


**Figure S3.** Concentrations of PFOS, fluoride ions, and sulfate ions during photodegradation (6-h) in non-buffered aqueous solution in Reactor B. Initial pH: 7.4, final pH: 4.3, temperature: ~90 °C.


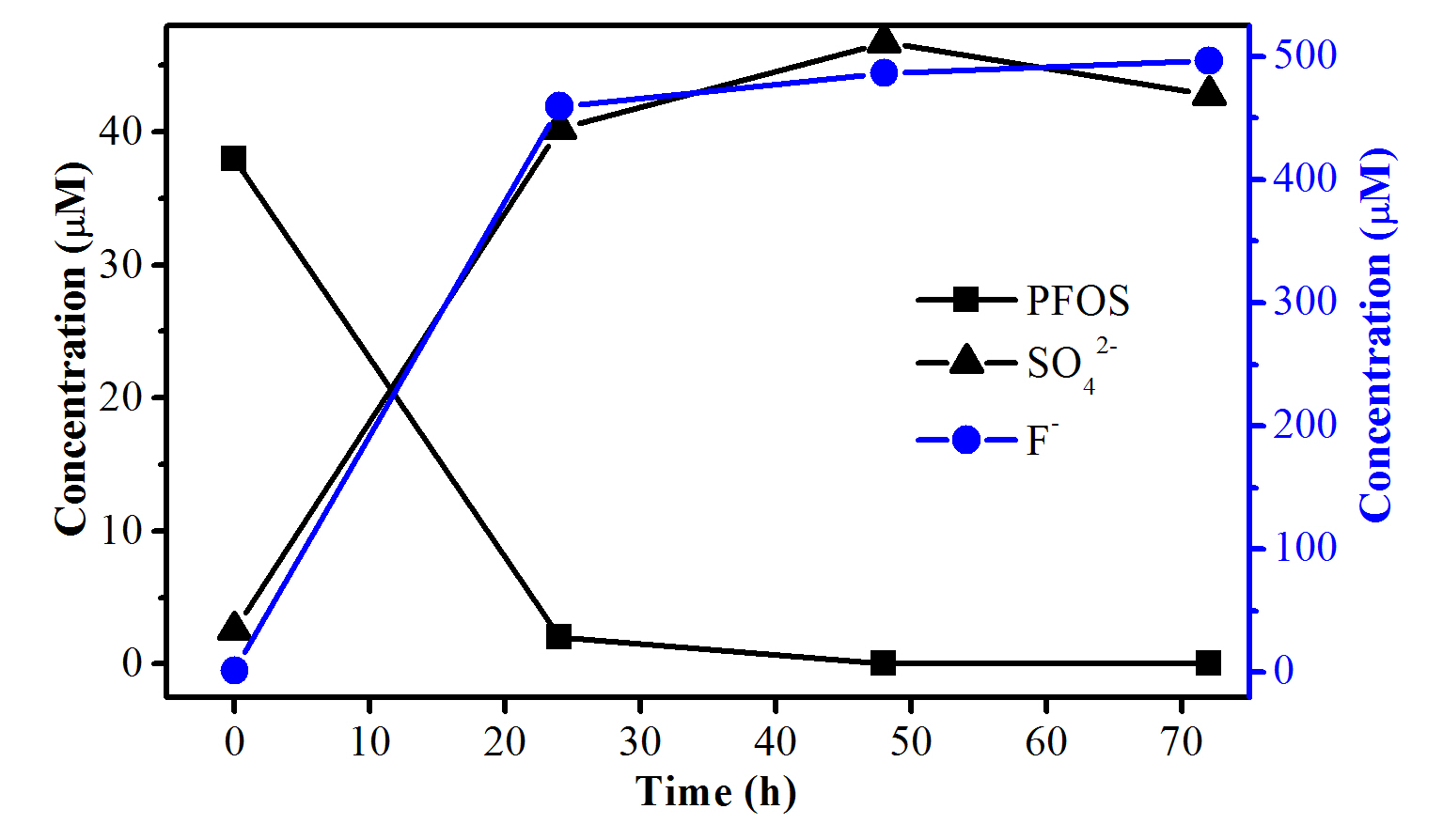


**Figure S4.** Concentrations of PFOS, fluoride ions, and sulfate ions during photodegradation in Reactor B in K_2_HPO_4_ (10.0 mM) solution; initial pH: 9.0, final pH: 7.9, temperature: ~90 °C.

**Figure S5.** Arrhenius plot of reaction rates for PFOS (37.2 μM) photodegradation in 6.0 mM PBS (pH 7.0). PFOS decomposition rate constant at 35 °C was excluded owing to its negative value.

**Figure S6.** The time profiles of PFOA (40 μM) decomposition and the fitted curve in PBS (6.0 mM, pH 7.0) at 90 °C.


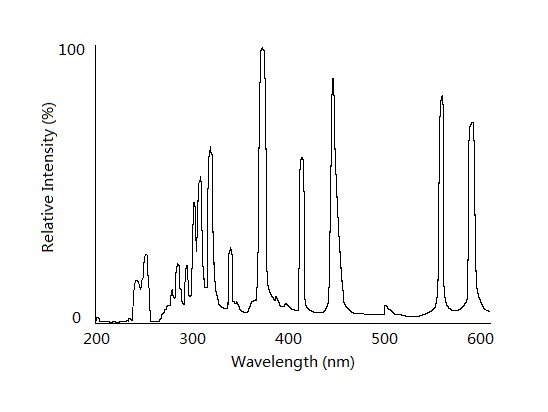
**Figure S7**. Wavelength distribution of emission from the medium pressure mercury lamp (data supplied by the manufacturer)


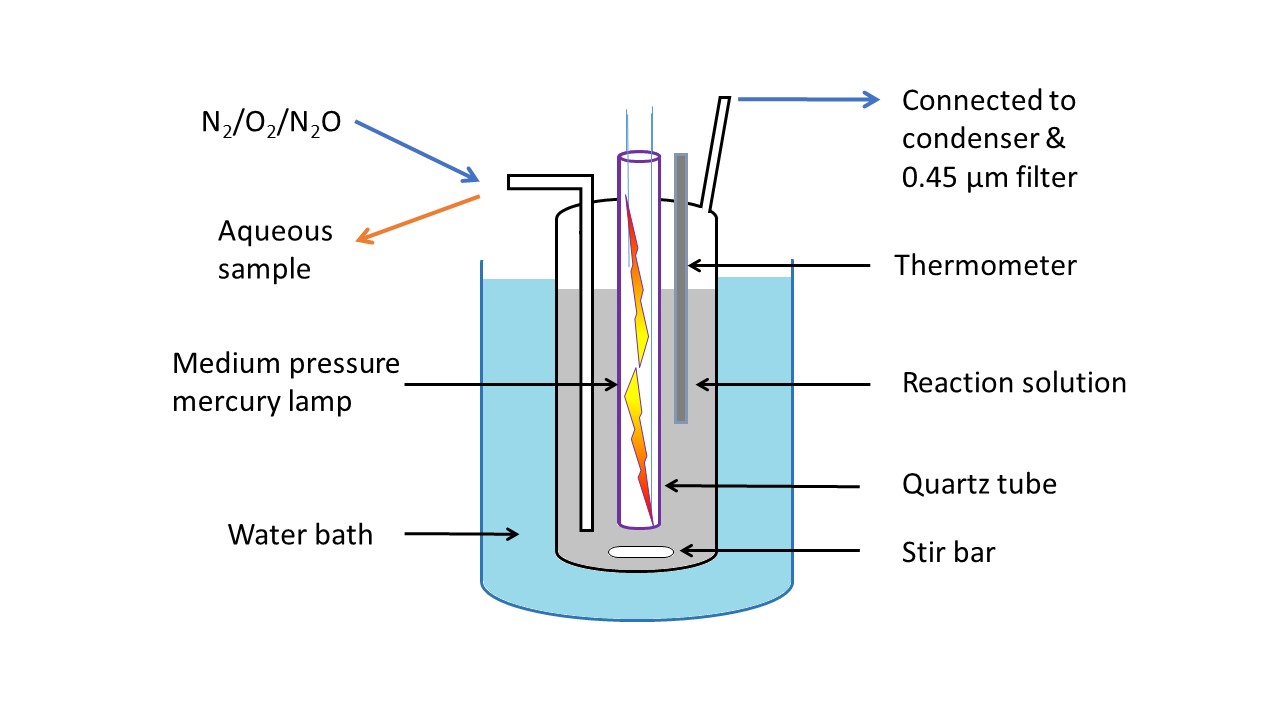


**Figure S8.** Schematic of the PFOS photodegradation system (Reactor A)

**Table S1. The fitted results of pseudo-ﬁrst-order decomposition rate constants of PFOS (37.2 μM) and defluorination ratios at various pHs in 6.0 mM PBS.**

| Initial pH | Final pH | *k* (h^-1^) | Standard Error | Adjusted R^2^ | Defluorination Ratio (6-h) |
| --- | --- | --- | --- | --- | --- |
| 2.4 | 2.3 | 0.00614 | 0.00445 | -0.2486* | -1.8579* |
| 3.1 | 3.1 | 0.02096 | 0.00274 | 0.6112 | 0.2697 |
| 4.1 | 3.9 | 0.03590 | 0.00268 | 0.8894 | 0.5456 |
| 5.0 | 4.5 | 0.06908 | 0.00488 | 0.9315 | 0.5888 |
| 6.0 | 5.9 | 0.09834 | 0.00712 | 0.9087 | 0.7567 |
| 7.0 | 6.9 | 0.16261 | 0.01415 | 0.8891 | 0.8216 |
| 8.0 | 7.7 | 0.14033 | 0.00859 | 0.9449 | 0.9336 |
| 9.1 | 8.1 | 0.13458 | 0.01237 | 0.8620 | 0.8989 |
| 10.1 | 8.3 | 0.13484 | 0.00952 | 0.9246 | 0.8418 |
| 11.0 | 9.7 | 0.15318 | 0.00681 | 0.9752 | 0.8134 |
| 11.8 | 11.4 | 0.11940 | 0.00365 | 0.9880 | 0.8613 |

*Negative value: probably because of no obvious degradation and measurement error.

**Table S2. The fitted results of pseudo-ﬁrst-order decomposition rate constants of PFOS (37.2 μM) and defluorination ratios under various temperatures in 6.0 mM PBS.**

| Temperature | Initial pH | Final pH | *k* (h^-1^) | Standard Error | Adjusted R^2^ | Defluorination Ratio (5-h) |
| --- | --- | --- | --- | --- | --- | --- |
| 35 ± 1 °C | 7.1 | 7.0 | -0.00030* | 0.00361 | 0.0013 | 0.1722 |
| 50 ± 1 °C | 7.0 | 7.0 | 0.02503 | 0.00177 | 0.9559 | 0.2336 |
| 60 ± 1 °C | 7.0 | 7.0 | 0.03306 | 0.00126 | 0.9762 | 0.3372 |
| 75 ± 1 °C | 7.0 | 7.0 | 0.04343 | 0.00407 | 0.8754 | 0.3494 |
| 90 ± 1 °C | 7.0 | 7.0 | 0.05826 | 0.00298 | 0.9646 | 0.7282 |
| 90 ± 1 °C | 11.8 | 11.6 | 0.07157 | 0.00282 | 0.9851 | 0.5976 |
| 95 ± 1 °C | 7.0 | 7.0 | 0.07256 | 0.00317 | 0.9656 | 0.8162 |
| 95 ± 1 °C | 11.8 | 11.6 | 0.11843 | 0.00361 | 0.9880 | 0.6324 |
| 100 ± 1 °C | 7.1 | 7.0 | 0.09450 | 0.00354 | 0.9830 | 0.7595 |
| 100 ± 1 °C | 11.8 | 11.4 | 0.91499 | 0.20176 | 0.9381 | 0.8833 |

*Negative value: probably because of no obvious degradation and measurement error.

**References**

1. Ohsawa, I. *et al.* Hydrogen acts as a therapeutic antioxidant by selectively reducing cytotoxic oxygen radicals. *Nat Med* **13**, 688-694 (2007).

2. Liu, G. M., Zhao, J. C. & Hidaka, H. ESR spin-trapping detection of radical intermediates in the TiO2-assisted photo-oxidation of sulforhodamine B under visible irradiation. *J Photoch Photobio A* **133**, 83-88 (2000).
